# Role of Vitamin D Status in Diabetic Patients with Renal Disease

**DOI:** 10.3390/medicina55060273

**Published:** 2019-06-13

**Authors:** Guido Gembillo, Valeria Cernaro, Antonino Salvo, Rossella Siligato, Alfredo Laudani, Michele Buemi, Domenico Santoro

**Affiliations:** Unit of Nephrology and Dialysis, Department of Clinical and Experimental Medicine, University of Messina, Via Consolare Valeria 98, 125 Messina, Italy; guidogembillo@live.it (G.G.); valecern82@virgilio.it (V.C.); asalvo@hotmail.it (A.S.); rossellasiligato@gmail.com (R.S.); alfredo.laudani@gmail.com (A.L.); buemim@unime.it (M.B.)

**Keywords:** albuminuria, calcitriol, CKD, diabetes, diabetic nephropathy, podocytes, VDR, Vitamin D status

## Abstract

Diabetes mellitus (DM) poses a major public health problem worldwide, with ever-increasing incidence and prevalence in recent years. The Institute for Alternative Futures (IAF) expects that the total number of people with type 1 and type 2 DM in the United States will increase by 54%, from 19,629,000 to 54,913,000 people, between 2015 and 2030. Diabetic Nephropathy (DN) affects about one-third of patients with DM and currently ranks as the first cause of end-stage kidney disease in the Western world. The complexity of interactions of Vitamin D is directly related with progressive long-term changes implicated in the worsening of renal function. These changes result in a dysregulation of the vitamin D-dependent pathways. Various studies demonstrated a pivotal role of Vitamin D supplementation in regression of albuminuria and glomerulosclerosis, contrasting the increase of glomerular basement membrane thickening and podocyte effacement, with better renal and cardiovascular outcomes. The homeostasis and regulation of the nephron’s function are absolutely dependent from the cross-talk between endothelium and podocytes. Even if growing evidence proves that vitamin D may have antiproteinuric, anti-inflammatory and renoprotective effects in patients with DN, it is still worth investigating these aspects with both more in vitro studies and randomized controlled trials in larger patient series and with adequate follow-up to confirm the effects of long-term vitamin D analogue supplementation in DN and to evaluate the effectiveness of this therapy and the appropriate dosage.

## 1. Overview on Vitamin D

### 1.1. Vitamin D Metabolism

Vitamin D belongs to a family of steroid fat-soluble hormones with a wide range of pleiotropic functions [1]. Its essential role in promoting bone health and calcium homeostasis is well known; beyond these classical functions, burgeoning evidence has shown new health benefits associated with adequate vitamin D metabolism, such as the modulation of cellular growth and differentiation, the regulation of innate and adaptive immune functions [2], a protective effect against diabetes and cardiovascular disease [3] and a much broader range of biological features. Indeed, the active form of vitamin D, through its nuclear vitamin D receptor (VDR), modulates the expression of about 1500 genes in numerous cell types [4].

Much of the total vitamin D in the body is retained in fat stores and there are two main sources of vitamin D: sunlight exposure, which leads to cholecalciferol (vitamin D_3_) production and gastrointestinal absorption (mainly vitamin D2, ergocalciferol).

Sun is the major determinant of the vitamin D status, in fact this molecule is also known as “sunshine vitamin.” Vitamin D can be produced endogenously through an ultraviolet (UV) light-dependent reaction: the cholesterol is converted, by 7-dehydrocholesterol reductase, in the precursor previtamin D, also known as 7-dehydrocholesterol. This precursor, through the action of UV rays on the skin, is changed into cholecalciferol, a process that requires up to three days [5].

Cholecalciferol and ergocalciferol need further metabolism to be activated. These two inert molecules must undergo a first hydroxylation on carbon 25 in the liver, in the endoplasmic reticulum of hepatocytes, where the 25-hydroxyvitamin D (calcidiol) is produced by the cytochrome P450 (CYP) enzyme Liver-25-hydroxylase (CYP2R1) [6]. The second hydroxylation is performed in the kidney, in the mitochondria of the proximal convoluted tubules: here the calcidiol is hydroxylated by 1-alpha-hydroxylase (CYP27B1) producing the 1,25-dihydroxy vitamin D, the active form of vitamin D, calcitriol.

The effects of calcitriol are primarily mediated by VDR, also known as calcitriol receptor or NR1I1 (nuclear receptor subfamily 1, group I, member 1) [7].

When occupied by calcitriol, the VDR genomic pocket interacts with the retinoid X receptor to form a heterodimer that binds to vitamin D responsive elements in the region of genes directly controlled by calcitriol. Activated VDR modulates the transcription of genes encoding proteins that carry out the traditional genomic functions of vitamin D, by recruiting complexes of either coactivators or corepressors [8,9].

The gene that provides instructions for synthesizing the 1 alpha-hydroxylase enzyme is the CYP27B1. In humans it is located on chromosome 12, 12q14.1, flanked by the genes for METTL21B (methyltransferase-like 21b), METTL1 (methyltransferase-like 1), MARCH9 (a member of the E3 ubiquitin ligase family), CKD4 (cyclin D kinase isoform 4) and mir6759 (a microRNA). The gene contains nine exons, including a very large exon 9 [10].

CYP27B1 is linked with the tightly controlled feedback cycle that leads to the synthesis of calcitriol by the double control of two hormones: parathyroid hormone (PTH) and fibroblast growth factor-23 (FGF23). PTH stimulates vitamin D production through increased transcription of the gene CYP27B1. Conversely, FGF23 induces down-regulation of the synthesis of calcitriol since it is not only the most important phosphaturic hormone but also decreases calcitriol synthesis through CYP27B1 and the stimulation of the mitogen-activated protein kinase pathway in the parathyroid glands, which decreases gene expression and the secretion of PTH with a following reduction in calcitriol synthesis [11].

In klotho null mouse, despite very high FGF23 levels, circulating calcitriol is very high, which questions that FGF23 is a major suppressor of 1-hydroxylase [12].

Most parts of the products of vitamin D catabolism are excreted through the bile into the faeces; the terminal step of calcitriol degradation is calcitroic acid (1α-hydroxy-23-carboxy-24,25,26,27- tetranorvitamin D), which is water-soluble and is excreted primarily in the urine [13]. In patients with preserved renal function, most of the vitamin D metabolites interact with the Vitamin D Binding Protein (DBP) through the cubilin–megalin receptor system [14].

### 1.2. Biomarkers of Vitamin D Status

The main indicator of vitamin D status is given by the level of serum 25(OH)D_3_ concentration, since it is the most abundant and stable vitamin D metabolite in human serum and has a half-life of 2–3 weeks. The half-life of 1,25(OH)_2_D is short (within hours) and most of all its formation is not directly regulated by vitamin D intake: even in the presence of severe vitamin D deficiency, 1,25(OH)_2_D levels may be in the normal range or elevated as a result of up-regulation of the 1α-hydroxylase enzyme [15]; conversely, 1,25(OH)_2_D_3_ circulates in the serum at very low concentrations: about 0.1% of those of the prohormone 25(OH)D_3_ [16].

With the aim to evaluate the most appropriate method to estimate the right concentration of vitamin D, several studies have systematically investigated emerging biomarkers of vitamin D status including the vitamin D metabolite ratio (ratio between 25-OHD and 24,25-dihydroxy vitamin D), bioavailable 25-OHD (25-OHD not bound to DBP) and free 25-OHD (circulating 25-OHD bound to neither DBP nor albumin) [17] but their role is still open to debate. It is quite right to emphasize that the most critical limitation of mass spectrometry, used to discriminate between circulating concentrations of 25-hydroxyvitain D and 24,25-dihydroxyvitamin D is that it is not available in all clinical laboratories.

### 1.3. Vitamin D Serum Level and Supplementation

The central role of an appropriate vitamin D metabolism is increasingly being investigated by the most important scientific societies: scientists worldwide have posed the problem about what is the optimal status of this basilar dietary component. These findings might offer new perspectives and answers to unresolved questions in understanding the human metabolic complexity. In the last five years more than 21,000 scientific articles have been published evaluating the properties of vitamin D [18].

The analysis elaborated by the Institute of Medicine (IOM) for recommendations for Vitamin D adequacy indicated a value <30 nmol/L (<12 ng/mL) as associated with vitamin D deficiency, leading to rickets in infants and children and to osteomalacia in adults, 30 ≤ 50 nmol/L (12≤20 ng/mL) was considered inadequate for bone and overall health in healthy individuals and ≥50 nmol/L (≥20 ng/mL) considered adequate for bone and overall health in healthy subjects [19].

The USA Endocrine Society subsequently upgraded these three thresholds to <20 ng/mL (50 nmol/L), 20–30 ng/mL (50–75 nmol/L) and 30–100 ng/mL (75–250 nmol/L), respectively (Table 1).

In accordance with these parameters, it has been estimated that 20–100% of U.S., Canadian and European elderly men and women are vitamin D deficient [20,21,22,23].

The National Kidney Foundation in the Kidney Disease Outcomes Quality Initiative (K/DOQI) clinical practice guidelines for bone metabolism and disease in chronic kidney disease (CKD) [24] declare that a 25D serum level of 30 ng/mL is considered as the threshold for vitamin D normal status and insufficiency.

In line with these findings, the Endocrine Society in the USA recommends achieving serum 25(OH)D concentrations of more than 30 ng/mL in adults. It suggests that all adults who are vitamin D-deficient should be treated with 50,000 IU of vitamin D_2_ or vitamin D_3_ once a week for 8 weeks or its equivalent of 6000 IU of vitamin D_2_ or vitamin D_3_ daily to achieve 25(OH)D blood levels above 30 ng/mL, followed by maintenance therapy of 1500–2000 IU/day [25] (Table 2).

The National Osteoporosis Society also suggests a level <30 nmol/L as deficient, 30–50 nmol/L as insufficient and >50 nmol/L as adequate [26].

A recent study published on The Lancet systematically reviewed meta-analyses of vitamin D supplementation and non-skeletal disorders published between 1 January 2013 and 31 May 2017, demonstrating the importance of maintaining the right vitamin D serum level and how its depletion may lead to a worsening of many adult conditions: the results also showed that the effect of vitamin D supplementation had a significantly greater impact in people with low 25(OH)D at baseline compared with other participants [27].

There is also increasing evidence of the significant influence of proper vitamin D status on delaying death in populations with specific risk factors: in a meta-analysis published in 2014, Bolland and colleagues [28] focused on the association between the serum level of vitamin D in patients with myocardial infarction or ischaemic heart disease (9 trials, 48,647 patients), stroke or cerebrovascular disease (8 trials, 46,431 patients), cancer (7 trials, 48,167 patients) and total fractures (22 trials, 76,497 patients) and mortality. The Authors found as secondary endpoint a reduction in all-cause mortality of 4%, showing that vitamin D supplementation is associated with substantially longer life expectancy in middle-aged and older adults.

## 2. Role of Vitamin D in CKD Patients

The central role of the synthesis of calcitriol in the kidney is well documented: dysfunctions in vitamin D metabolism, ubiquitous in patients with CKD, are a key feature of both skeletal and non-skeletal complications in all the tissues where VDR has been identified, such as parathyroid glands, intestine, immune system and have been associated with all-cause mortality and cardiovascular events [29,30,31,32].

The regulation of vitamin D level is intriguing and controversial, beside the central role of 1,25(OH)_2_D-24-hydroxylase (CYP24A1) to control its status [33], even the hormone 1,25-(OH)_2_D_3_ itself is established as a role as suppressor of 1α-hydroxylase activity [34]. Moreover, the availability of 1*α*-hydroxylase is reduced as kidney mass declines [35]. As complicated as it is to maintain a good level of vitamin D, the major role that it plays in the body homeostasis is undisputed: vitamin D concentration is directly related with progressive long-term changes implicated in the worsening of renal function and quality of life [36]. These changes result in a dysregulation of the vitamin D-dependent pathways.

The CKD patient is at high risk for Vitamin D deficiency for multiple causes. Among the principal reasons there are: the loss of the major carrier protein for 25(OH)D, the DBP, due to nephrotic-range proteinuria associated to type 2 diabetes and some types of glomerulonephritis; the severe restriction of food containing vitamin D to avoid an excess of phosphorus intake; the little time spent to sunlight exposure and most of all, the marked reductions in the renal proximal tubule expression of the endocytic receptor megalin. Decreases in renal megalin take place already at very early stages of CKD, this status lead to a decreased re-absorption of glomerular-filtered albumin and other low-molecular-weight-proteins, as demonstrated in the megalin null mice with normal renal function [37,38]. It is also well demonstrated that a close linkage exists between low levels of 25OHD and the development of cardiovascular disease due to the activation of the renin–angiotensin–aldosterone system (RAAS) [39,40]. 1,25-(OH)_2_D_3_ inhibits this system so reducing arterial blood pressure and cardiac hypertrophy and then morbidity and mortality in patients with cardiorenal syndrome [41,42,43].

It is still on debate which is the right target of vitamin D for CKD patients and how to obtain the most effective dose that can lead to the desirable level. Recently, a Scientific Workshop sponsored by the National Kidney Foundation agreed that clinicians should classify 25(OH)D “adequacy” as concentrations > 20 ng/mL without elevated counter-regulatory PTH activity and that 25(OH)D concentrations < 15ng/mL should be treated independently of PTH level [44].

Numerous studies have explored the effects of different serum levels of vitamin D in patients with CKD.

In an analysis of the National Health and Nutrition Examination Survey (NHANES) examining the data of 14,679 participants it has been demonstrated that 25(OH) D concentrations were significantly lower only in the participants with a severe (15–29 mL/min/1.73 m^2^) decrease in eGFR compared to those with normal kidney function (61.6 vs. 73.3 nmol/L, *p* = 0.0063) [45].

As reported by K/DOQI clinical practice guidelines for bone metabolism and disease in CKD, 78% of new dialysis patients develop vitamin D deficiency [24]. Namyr et al. [46] investigated 516 CKD patients for a connection between vitamin D and the delaying of dialysis treatment. They observed an association between baseline 25(OH)D levels below 15 ng/mL and renal outcomes (start of dialysis or a rise of at least 50% in serum creatinine); they also found out the promising result that an increment of 10 ng/mL in 25(OH)D values was associated with a 25%-reduction in mortality, demonstrating that vitamin D depletion in CKD could be a convenient biomarker indicating patients in which adverse renal outcomes can be anticipated [47]. The meta-analysis performed by Duranton F. et al. [48], including data from 14 observational studies (194,932 patients), strengthened the hypothesis that receiving any vitamin D therapy significantly reduced the risk of all-cause mortality in patients with CKD receiving conservative care and in haemodialysis patients, with a relative risk of death that was 0.72 (95% CI 0.65–0.80) after 3 years of therapy and 0.67 (95% CI 0.45–0.98) after 5 years.

The mortality rate was even valuated in a large historical cohort study. Teng et al. [49] analysed the data of 51,037 incident haemodialysis patients between January 1996 and December 2002 who survived for at least 90 days from the initiation of haemodialysis. They demonstrated that the mortality rate at two years was lower in the group receiving injectable activated vitamin D (13.8/100 person-per-year) compared to the group that did not (28.6/100 person-per-year, *p* < 0.001). A recent meta-analysis conducted by Lu Rj et al. [50] investigated the effects of vitamin D or its analogues on the mortality of patients with CKD, evaluating the data of 38 studies involving 223,429 patients [(17 randomized controlled trials (RCTs), (*n* = 1819); (21 observational studies, *n* = 221,610)]. Vitamin D supplementation was significantly associated with a 38% reduction in all-cause mortality and 45% reduction in cardiovascular mortality in observational studies; even if RCTs did not provide sufficient or precise evidence that vitamin D supplementation affects mortality of patients with CKD, this underline the fact that more studies are needed to find stronger results.

The evidence enlightening the role of vitamin D to prevent worsening of CKD can be explained by a plethora of regulating effects that can reduce the risk of renal damage, such as the modulation of the response to metabolic disorders and the control of the progressive deterioration or irreversible damage of renal structures and functions. The right maintenance of vitamin D represents a pivotal strategy to prevent the complications that worsen with progression of the disease, therefore additional efforts to validate the proper modulation of the factors influencing vitamin D are needed.

## 3. Vitamin D and Diabetes in Kidney Diseases

### 3.1. Vitamin D Status in Diabetic Patients with Different Stages of Renal Disease

Diabetes mellitus (DM) poses a major public health problem worldwide, with ever-increasing incidence and prevalence in recent years. The Institute for Alternative Futures (IAF) expects that the total number of people with type 1 and type 2 DM in the United States will increase by 54%, from 19,629,000 to 54,913,000 people, between 2015 and 2030 [51,52].

Diabetic Nephropathy (DN) affects about one-third of people with DM [53] and currently ranks as the first cause of end-stage kidney disease (ESKD) in the Western world. The major clinical manifestations of DN are proteinuria, haematuria and progressive reduction of renal function: United States Renal Data System registered that approximately 35–50% of ESKD cases are secondary to DN complications [54]. Notwithstanding all the attempts made to configure a definitive strategy to slow-down the progression of renal damage in DN patients, the results are still controversial. A pivotal role for the management of DM could be found in the pleiotropic actions of vitamin D. Studies conducted by Afzal et al. [55], Schöttker et al. [56], Tsur et al. [57], have shown a link between low serum levels of vitamin D and the increasing risk of developing DM. M. Flores et al. [58] associated a proper status of vitamin D with a better modulation of glucose homeostasis, due to a regulation of insulin secretion and tyrosine phosphorylation of the insulin receptor. De Boer et al. [59] analysed the data of the Third National Health and Nutrition Examination Survey (NHANES III) and found an inverse association between the level of vitamin D and the prevalence of albuminuria. The study of Karnchanasorn et al. [60] showed another modulatory effect of vitamin D: in particular, the authors demonstrated a correlation between appropriate vitamin D status and both β-cell function and insulin sensitivity.

On the other hand, as important as it is to maintain a proper status of 25(OH)D, many studies showed how its value is substantially decreased in patients with DN.

In the study of Navaneethan D. et al. [61] the data of 2403 diabetic patients with stage 3-4 CKD have been examined to evaluate factors associated with low 25(OH)D levels and all-cause mortality; this cohort has been divided in three groups (patients with vitamin D level <15, 15–29 and ≥30 ng/mL). The group that had insufficient vitamin D concentrations (<15 ng/mL), made by 777 patients (32.33%), had a 53%-increased risk of mortality compared to patients with vitamin D values ≥30 ng/mL with a hazard ratio (HR) for all-cause mortality between these two groups of 1.33 (95% CI, 1.07–1–65).

Wahl et al. [62] analysed the data of patients with stage 2–4 CKD retrieved from the Chronic Renal Insufficiency Cohort Study: they found that participants with DM (n = 1.820) had significantly lower vitamin D levels (23.9 ± 13.3 ng/mL) compared to those without diabetes (*n* = 1.936, vitamin D 31.0 ± 14.8 ng/mL; *p* < 0.001).

In 2015, Peng et al. [63] performed a study on 448 patients, of which 144 with type 2 DM and CKD and 304 with CKD without DM: their analysis showed an insufficient vitamin D status for 93.1% of DN patients and 78.9% of patients without DN, with a median value for the DN group of 8.5 ng/mL (interquartile range (IQR) 6.8–11.3 ng/mL), a level significantly lower than the control group [13.9 ng/mL, IQR 11.2–18.2 ng/mL, *p* < 0.0001]. Moreover, the Authors found that the determination of 25(OH)D status might be employed to identify patients at increased risk of developing renal complications and that 25(OH)D could be used as an indicator for diagnosis of DN with the cut-off value of 10.5 ng/mL (a 6.559-fold increased risk of DN).

In a retrospective study performed by Sipahi S. et al. [64] that enrolled 1463 patients, 770 of them (52.0%) had a baseline serum level of vitamin D <20 ng/mL. Among them, 383 patients had vitamin D supplementation (75.000 units weekly); after an average of 5 months, there was a significant increase of 25(OH)D levels (from a median level of 18.1 ng/mL pre-treatment to 25.5 ng/mL post-treatment), in agreement with previous studies [65,66]. The Authors also revealed a significant role of HbA1c in predicting a decrease in vitamin D levels. Moreover, the median HbA1c at baseline was higher in patients deficient in vitamin D than in those with a normal vitamin D level (7.3 IQR 3.9 vs. 6.5, IQR 2.3%; *p* < 0.01); this ability to lower the concentration of this DM biomarker has also been demonstrated in other studies on DM patients with CKD [67,68,69].

In a retrospective observational study conducted by Xiao et al. [70], 240 patients with DN were sequentially grouped according to CKD stage and compared with 60 control subjects. The serum 25(OH)D levels of the four DN groups (CKD 5 7.74 ± 2.90, CKD 4 8.44 ± 2.53, CKD 3 10.31 ± 3.36, CKD 1-2 12.23 ± 4.07 ng/mL) were significantly lower than in the control group (29.43 ± 10.15 ng/mL; *p* < 0.05); moreover, as reported by previous studies, the serum 25(OH)D deficit was associated with higher fasting blood glucose and HbA1c.

A recent article by Ray et al. [71] investigated the profile of CKD related-mineral bone disorders in 72 newly diagnosed DN patients with CKD stage 4 and 5. Vitamin D level was <20 ng/mL in 65.72% of them. In the CKD stage 4 group serum 25(OH)D was 19.15 (IQR 13.6-23.4) ng/mL, whereas in the CKD stage 5 group it was 10.95 (IQR 9.3, 16.4) ng/mL (*p* = 0.006); the Authors also observed a significant negative correlation between urine albumin-to-creatinine ratio (ACR) and 25(OH)D (*p* = 0.002) (Table 3).

### 3.2. Role of Vitamin D Supplementation in Patients with Diabetes and CKD

As stated above, patients with DN have a higher probability to have suboptimal vitamin D levels: this condition can lead to a faster progression of both DM and CKD.

This status for DN patients is often caused by inadequate dietary intake of vitamin D due to restrictions on foods and beverage as these aliments also have a relevant carbohydrate, phosphorus and/or potassium content.

In case of not enough food intake of vitamin D or limited sunlight exposure, a supplementation with vitamin D should be considered. In the United States and Canada, milk is fortified with vitamin D, as are some bread products, orange juices, cereals, yogurts and cheeses.

In Europe, most countries do not fortify milk with vitamin D because in the 1950s, there was an outbreak of vitamin D intoxication in young children, resulting in laws that made further restriction ruling the fortification of foods with vitamin D. Currently Sweden and Finland fortify milk and many European countries add vitamin D to cereals, breads and margarine [72].

Form, doses and schedule that should be used to achieve the best results are still on debate. Moreover, there is not strong evidence on a clinical threshold value for vitamin D deficiency or sufficiency with reference to DN [73].

Vitamin D has been proposed to have a relevant role into glucose and insulin homeostasis and, if low, as a risk factor for diabetes onset and progression. This aspect was shown in the meta-analysis of Tang et al. [74] that included 47 RCTs and involved 44,161 nondiabetic individuals with a median trial duration of 4 months and a median vitamin D dose of 4000 IU/d. In this study, vitamin D supplementation significantly reduced fasting glucose by 0.11 mmol/L, fasting insulin by 1.47 mIU/L and HOMA-IR (Homeostatic Model Assessment for Insulin Resistance) by 0.32 while increasing total 25 (OH) D levels by 40.14 nmol/L. In the double-blind, placebo-controlled RCT performed by Niroomand et al. [75], it was proved that high-dose vitamin D improves insulin sensitivity and decreases the risk of progression toward DM in a population of adults with pre-diabetes and vitamin D deficiency.

A protective role of vitamin D in type 1 DM has been demonstrated in a recent study by Panjivar et al. [76], where serum 25-(OH) D concentrations sustained with cholecalciferol supplementation 3000 IU/day for one year improved metabolic control and slowed the decline of residual β-cell function in children with T1DM. The meta-analysis by Gregoriou et al. [77] also showed that vitamin D supplementation in the form of 1α(OH)vitamin and cholecalciferol appears to be beneficial in the treatment of T1DM patients by attenuating the natural history of the disease.

Mager et al. [78] compared the impact of daily (2000 IU) versus monthly (40,000 IU) vitamin D_3_ supplementation over six months on markers of vitamin D status; the authors observed equivalent adherence and improvements in overall vitamin D metabolism, with an increase in serum 25(OH)D of 19 (12–26) nmol/L (*p* < 0.001) compared to baseline.

In the RCT of Barzegari et al. [79] DN patients with marginal serum vitamin D have been treated with 1.25-dihydroxycholecalciferol (50,000 IU/week) for 8 weeks. Vitamin D supplementation significantly increased vitamin D levels in the intervention group; a significant reduction in the serum levels of triglycerides, low density lipoproteins and total cholesterol (*p* = 0.04, *p* = 0.006 and *p* = 0.02, respectively) has also been reported in treated patients.

Another emerging issue linked with DN development and lower levels of vitamin D is urinary megalin excretion in T2DM patients, well demonstrated in the study of Ogasawara et al. [80]. Megalin, as a member of the low-density lipoprotein receptor family [81], plays a pivotal role in the reabsorption of vitamin D binding protein from glomerular filtrates [82]. Kuwahara et al. [83] reported that megalin-mediated (auto)lysosomal dysfunction in primary tubular epithelial cells is decisive for the development of kidney disease in a High-Fat-Diet-induced diabetes model. De S. et al. [84] found that exocytosis-mediated urinary megalin excretion increases along with the progression of DN, giving further contributions in the understanding of the pathogenesis of vitamin D loss in these subjects, their findings also suggest a potential role of megalin urinary excretion as indicator of progression of DN.

Although vitamin D is widely recognized as renal protective in DN, the exact dosage of its supplementation that should be administered to reach adequate serum levels and which is the appropriate vitamin D status in these patients remain controversial and more studies are needed to achieve stronger evidence.

### 3.3. Role of Vitamin D in Modulation of RAAS and Microalbuminuria in DN Patients

Progression of diabetic renal disease and increased cardiovascular risk factors such as raised blood pressure, dyslipidaemia and endothelial activation in DM are strictly correlated to the increase of urinary albumin excretion [85,86]. It has become increasingly clear that regression of albuminuria is linked with better renal and cardiovascular outcomes secondary to DN.

Various studies demonstrated that reduction of urinary albumin excretion by inhibition of the RAAS is associated with the preservation of renal function [87,88,89,90]. The systemic RAAS components are downregulated in DM [91], while renal interstitial angiotensin II levels are 1000-fold higher than in the plasma assuming a relevant damaging action [92]. The intrarenal angiotensin II has different roles in the worsening of CKD, causing renal inflammation that leads to renal cortical damage, increasing glomerular capillary pressure and permeability and subsequent proteinuria, along with alterations in renal hemodynamics [93,94].

Many studies demonstrated a better reduction in albuminuria with the association of RAAS inhibitors and vitamin D treatment [95,96,97].

Notwithstanding promising findings of observational studies and animal experimentations, there are few RCTs investigating the beneficial effects of vitamin D supplementation in patients with DN or the results are negatively influenced by the short duration of the follow-up. In literature there are just 3 RCTs on vitamin D supplementation in DN with a follow-up of at least 24 weeks but their outcomes highlighted encouraging results in terms of albuminuria control.

The RCT performed by De Zeeuw et al. [98], enrolling patients with type 2 DM (T2DM) and albuminuria treated with angiotensin-converting enzyme inhibitors or angiotensin receptor blockers, demonstrated the relevant efficacy of paricalcitol in reducing urinary albumin-to-creatinine ratio (UACR) in diabetic patients receiving 1 μg/day or 2 μg/day paricalcitol compared to controls. Indeed, the control group had −3% change in UACR (from 61 to 60 mg/mmol; 95% CI, −16 to 13), whereas the overall paricalcitol groups had −16% change in UACR (from 62 to 51 mg/mmol; 95% CI, −24 to −9). Another study that underlined the efficacy of the association of vitamin D supplementation and drugs acting on the RAAS is the one conducted by Tiryaki et al. [99]. The daily supplementation of 0.25 mcg calcitriol for 24 weeks induced a significant reduction in UACR (from 186.58 ± 22.22 to 142.72 ± 12.38 mg/g, *p* < 0.014) and urine angiotensinogen/urine creatinine ratio (UAGT/UCre) compared to baseline (from 12.96 ± 2.76 to 8.64 ± 2.24 mg/g, *p* < 0.012). In the RCT of Liyanage et al. [100], patients received intramuscular administration of 50,000 IU (0.25 mL) of vitamin D_3_ monthly for 6 months; the mean reduction of UACR was 51.8 mg/g (95% CI, −66.1–−37.5, *p* ≤ 0.001) in the treatment group, 22.4 mg/g (95% CI; −45.7–0.8, *p* = 0.06) in the control group and this difference was statistically significant (*p* = 0.001); a significant inverse correlation was observed between vitamin D values and percentage change in plasma renin levels (*r* = −0.66, *p* < 0.01) and percentage change in UA levels (*r* = −0.47, *p* < 0.01). Another relevant result was the mean reduction in plasma renin in the treatment group, attested to −5.85 pg/mL (95% CI; −6.7–−4.6, *p* < 0.001), (suggesting another possible renoprotective effect of vitamin D: this outcome has been previously reported in animal studies [101] (Table 4).

The reason of this effect could be found in the inhibition of renin gene promoter operated by the activated form of vitamin D through modulation of the action of liganded-VDR, which blocks the binding of CRP binding protein (CBP) at renin promoter thus preventing renin transcription [102,103].

The interaction of plasma renin and vitamin D is tightly connected with the VDR status: in case of vitamin D deficiency there is a reduced transcription of VDR and an enhanced degradation of unliganded VDR, with a decrease in both unliganded and liganded VDR. This deficiency of liganded VDR, as previously mentioned, would improve the transcription of renin whereas the lack of unliganded VDR would enhance the transcription of angiotensinogen and Angiotensin II Type I Receptors (AT1Rs) via modulation of p53 expression, as proved in an animal experiment conducted by Chandel et al. [104]. Thus, it appears that lack of VDR or VDR deficient state has at least two ways to activate the RAAS and vitamin D could be a modulator of RAAS through VDR control. Zhang et al. [105] tested the link between VDR deficiency and hyperglycaemia-induced renal injury in mice. In their study, diabetic VDR-knockout mice developed more severe albuminuria and glomerulosclerosis due to increased glomerular basement membrane thickening and podocyte effacement; the Authors also found a correlation between lack of VDR and increased values of renin, angiotensinogen, transforming growth factor-β and connective tissue growth factor.

Humalda et al. [106] underlined that the paradoxical event secondary to the use of RAAS inhibitors is the compensatory local renin rise, termed as aldosterone escape. To contrast this eventuality, it could be decisive to antagonize the renin activity with the use of 1,25(OH)_2_D_3_. They performed a systematic review of all RCTs with calcitriol or paricalcitol as an antiproteinuric intervention. During follow-up, active vitamin D analogues reduced proteinuria on average by 16%: these results were obtained in the majority of cases (84% overall) in patients under pre-existing chronic treatment with RAAS inhibitors, accentuating the ability of vitamin D analogues to reduce residual proteinuria [107].

### 3.4. Role of Vitamin D in Endothelium and Podocyte Preservation in DN

The progression of DM is strictly connected with oxidative stress [108] and functional and anatomical abnormalities of endothelial cells, glomerular basement membrane and podocytes that lead to an increased vascular permeability, accelerated atherosclerosis and proteinuria. Hyperglycaemia in DN is responsible for the retraction and flattening of podocytes’ foot processes [109], with a significant reduction of their number [110] and differentiation [111] and consequent failure to maintain their function in the integrity of the glomerular filtration barrier. The important role that podocyte injuries play in the development and progression of DN is given by the evidence coming from renal biopsies: indeed, it has been demonstrated that, already at the stage of albuminuria, ultrastructure of podocytes is altered in T2DM patients with and without DN [112].

Recent studies suggest that podocytes can be the direct target of circulating hormones, lipids, adipokines and insulin in diabetes [113,114]. The homeostasis and regulation of the nephron function are absolutely dependent from the cross-talk between endothelium and podocytes: in fact, podocytes synthesize the vascular endothelial growth factor (VEGF) that interacts with the receptors VEGFR-1 and VEGFR-2 expressed on the glomerular endothelium [115]. Another example is represented by the role of the activated protein C (APC), a protein regulated by the endothelial thrombomodulin that is a proteoglycan highly represented in the endothelial glycocalyx with a role in the modulation of haemostasis and endothelial response to inflammation. APC, which is reduced in DN patients, prevents the apoptosis of both endothelial cells and podocytes, an action that provides protection from the development of DN [116]. Wu-Wong et al. [117] evidenced that paricalcitol and calcitriol are both able to modulate the thrombomodulin expression in human aortic smooth muscle cells. Moreover, D’Arrigo et al. [118] demonstrated a raise of soluble thrombomodulin levels in patients with CKD by the administration of paricalcitol; they also found that such an effect is rapidly reversible after stopping the treatment. Indeed, thrombomodulin returned to baseline values 2 weeks after the last dose of the drug.

The preservation of the endothelial function is very crucial to delay the progression of kidney impairment and to prevent dangerous life-threatening effects. The association between vitamin D status and vascular function in patients with CKD has been demonstrated in several observational studies [119,120]. Endothelium preservation has been studied in the meta-analysis (seven RCTs, n = 429 patients) performed by Dou D. et al. [121], which investigated the ability of vitamin D supplementation to improve vascular function in patients with CKD. They found that inactive vitamin D had cardiovascular benefits compared to placebo: in particular, cholecalciferol significantly improved the Flow-Mediated Dilatation parameter (FMD) (weighted mean difference [WMD] 5.49%; 95% CI 4.36–6.62, *p* < 0.0001), an essential marker providing independent prognostic information in CKD patients; in the 1 ug paricalcitol group, there was no significant difference between the two groups (WMD − 0.22%; 95% CI −1.33 to 0.88, *p* = 0.69), In the 2 ug paricalcitol group, compared with the placebo group, paricalcitol significantly increased FMD (WMD 2.09%; 95% CI 1.28–2.9, *p* < 0.0001). This meta-analysis also showed that all types of vitamin D supplementation improved pulse-wave-velocity, another marker of arterial stiffness associated with variability of blood pressure and wall thickness, in CKD patients compared to placebo (WMD −0.93 m/s; 95% CI −1.71 to −0.15, *p* = 0.02; without significant heterogeneity, *p* = 0.14, I 2 = 45%).

More experiments on the vitamin D action on endothelial dysfunction have been performed by Vila Cuenca et al. [122], who demonstrated the ability of paricalcitol to protect against uraemia-induced endothelial damage by promoting vascular endothelial-cadherin-based cell-cell junctions and diminishing F-actin stress fibre organization: this prevents the formation of endothelial intracellular gaps. In the study of Zhang et al. [123] 71 non-dialysis CKD patients with low vitamin D (serum 25(OH)D < 30 ng/mL) received 50,000 units of oral cholecalciferol once a week for 12 weeks. At the end of the treatment period, they found a significant increase of the endothelial function by the measurement of the brachial artery FMD from 4.4 ± 1.3 to 5.1 ± 1.5% (*p* < 0.001) and a decrease of soluble endothelial biomarkers, such as soluble vascular cell adhesion molecule-1 (sVCAM-1) from 926.9 ± 158.0 to 867.0 ± 129.0 ng/mL (*p* < 0.001) and sE-selectin from 69.7 ± 15.8 to 63.3 ± 14.7 ng/mL (*p* < 0.001).

In the study by Deda et al. [124], 227 diabetic patients have been treated with cholecalciferol at doses of 1000 or 2000 IU daily for 12 to 24 weeks with an improvement in endothelial function measured by the change of reactive hyperaemia index (RHI). Zoccali et al. [125], in a double-blinded RCT on 88 patients, also demonstrated that paricalcitol improves endothelium-dependent vasodilatation in patients with stage 3 to 4 CKD. After 12 weeks of treatment, FMD incremented in the paricalcitol but not in the placebo group, the between-group difference in FMD changes was significant (1.8%, 95% CI, 0.3–3.1; *p* = 0.016) and the mean proportional change in FMD was 61% higher in paricalcitol-treated patients than in placebo-treated patients. The effect faded away 2 weeks after the treatment was stopped.

Recently Hammer et al. [126] demonstrated in vitro a direct action of active form of vitamin D on the Endothelial Progenitor Cells (EPCs), a population of bone marrow-derived cells that have a relevant part in the process of endothelialisation and vascular repair following an injury [127]. Impairment of EPCs, which occurs in patients with diabetes, was shown to be related to endothelial dysfunction, coronary artery disease and adverse clinical outcomes. In previous studies, even Yiu et al. [128] demonstrated an inverse relationship between serum vitamin D concentrations and circulating EPC levels in patients with DM.

### 3.5. Role of Vitamin D in the Prevention of Glomerulosclerosis and Inflammatory Processes in DN

Increasing evidence from experimental and clinical studies has unveiled a pathological role of macrophages in the development of glomerulosclerosis by the production of inflammatory chemokines, cytokines and fibrogenic factors, release of proteolytic enzymes and production of reactive oxygen species [129,130]. A central role in these mechanisms is played by the monocyte chemoattractant protein (MCP)-1 [131], a chemokine produced by mesangial cells (MCs) and renal tubular cells that has the responsibility of recruiting macrophages into the kidney [132]. Zhang et al. [133] demonstrated that calcitriol can inhibit the synthesis and activity of MCP-1 and contrast the glomerular injury in diabetic mice. Their data suggest that vitamin D may protect against renal injury in DN by preventing or reducing macrophage infiltration.

A proper vitamin D regulation also has an important role against inflammation secondary to DN. Indeed, diabetes leads to an increase in the expression of inflammatory factors and inappropriate immune activity [134]. Inflammatory response likely contributes to DM occurrence by worsening insulin resistance and it is in turn intensified in the presence of hyperglycaemia to exacerbate long-term complications of diabetes [135].

Vitamin D exerts protective effects against inflammatory agents by inhibiting the expression of interleukin (IL)-6, IL-8, RANTES (regulated on activation, normal T cell expressed and secreted), intercellular adhesion molecule-1 (ICAM-1), vascular cell adhesion molecule-1 (VCAM-1), platelet-endothelial cell adhesion molecule-1 (PECAM-1), receptor of advanced glycation end products (RAGE) and E-selectin through a nuclear factor-κB (NF-κB)–mediated mechanism, partly by disrupting DNA binding of NF-κB.

Moreover, vitamin D represses the expression of cyclooxygenase (COX)-2 and upregulates the expression of 15-hydroxyprostaglandin dehydrogenase (15PGDH), the enzyme initiating prostaglandin catabolism, in this way reducing prostaglandin levels and suppressing the production of several proinflammatory cytokines [136,137]. The immunomodulatory action of vitamin D has been well demonstrated also in the study of Lucisano et al. [138], where an acute paricalcitol supplementation induced a significant reduction of IL-17, IL-6, IL-1β, TNF-α and IFN-γ in a cohort of CKD patients.

In a 2018 study of Zheng et al. [139] involving 101 patients, the Authors investigated the expression of anti-inflammatory cytokine protein tyrosine phosphatase nonreceptor type 2 (PTPN2) in DM patients and the relationship with VDR. They found out that paricalcitol administration increased PTPN2 expression by promoting its anti-inflammatory activities in case of high stimulation with glucose.

In the study of Duncan et al. [140] using human incubated monocytes taken from patients with DM and DN with uraemia, it was found that vitamin D may exert an anti-inflammatory effect by regulating the signal transduction pathways that control VDR and signal transducer and activator of transcription 5 (STAT5) expression. These findings have been further investigated by Yang et al. [141], who analysed more anti-inflammatory effects of vitamin D and VDR on phosphorylated STAT5 (p-STAT5) in serum-incubated monocytes from patients with DM and uraemia caused by DN: lipopolysaccharide associated with IL-15 upregulated the expression of p-STAT5, whereas pre-treatment with 1,25-(OH)_2_D_3_ significantly inhibited this effect.

## 4. Conclusions

Experimental as well as observational studies and clinical trials conducted in the past years suggest the effective role of vitamin D and the synergic action with RAAS inhibitors to counteract the worsening of DN and to preserve the glomeruli and the integrity of glomerular filtration barrier; moreover, vitamin D seems to exert many extra-renal functions essential for the body homeostasis.

The results of these studies emphasize the need for better awareness among researchers and clinicians about the consequences of insufficient vitamin D levels and the importance of monitoring its status in high-risk populations.

Even if growing evidence proves that vitamin D may have antiproteinuric and renoprotective effects in patients with DN, it is still worth investigating these aspects with more RCTs in larger patient series and with adequate follow-up to confirm the effects of long-term vitamin D treatment and to evaluate the effectiveness of the therapy and the appropriate dosage.

## Figures and Tables

**Table 1:** Different grading of 25 hydroxyvitamin D status for the Institute of Medicine and USA Endocrine Society.

	Vitamin D Levels Associated to Deficiency	Vitamin D Levels Associated to Insufficiency	Adequate Vitamin D Levels
Institute of Medicine recommendations	<30 nmol/L (<12 ng/mL) ^a^	30 ≤ 50 nmol/L (12 ≤ 20 ng/mL)	≥50 nmol/L (≥20 ng/mL)
USA Endocrine Society recommendations	<50 nmol/L (<20 ng/mL)	50–75 nmol/L (20–30 ng/mL)	75–250 nmol/L (30–100 ng/mL)

^a^ (1 nmol/L = 0.4 ng/mL)

**Table 2:** Recommended dietary intakes and supplementations of vitamin D for patients at risk for vitamin D deficiency according to the USA Endocrine Society.

	Recommended Vitamin D Dietary Intake	Obese Patients or Patients on Anticonvulsant Medications, Glucocorticoids, Antifungals and Medications for AIDS	Vitamin D Supplementation to Rise the Blood Level of 25(OH)D above 30 ng/mL
19–50 yr	At least 600 IU/d	Double or triple dose	At least 1500–2000 IU/d of vit D
50–70 yr	At least 600 IU/d	Double or triple dose	At least 1500–2000 IU/d of vit D
70+ yr	At least 800 IU/d	Double or triple dose	At least 1500–2000 IU/d of vit D

**Table 3:** Vitamin D baseline status in DN patients with different values of GFR. Data are expressed as mean ± standard deviation or median (IQR).

Serum 25 (OH) Vit D Baseline Levels in Different Stages of CKD					
Study, [Reference], (Year)	Populations Characteristics: Adults with CKD (either Secondary to Type 1 or 2 Diabetes Mellitus)	eGFR <15 mL/min/1.73 m^2^ (Stage 5) Patients Number (n)	CKD eGFR 15-60 mL/min/1.73 m^2^ (Stage 3–4) Patients Number (n)	eGFR 60-90 mL/min/1.73 m^2^ (Stage 2) Patients Number (n)	eGFR <90 mL/min/1.73 m^2^ (stage 1) Patients Number (n)
Navaneethan Sankar D [61] (2011)	2403 (n) DM with stage 3–4 CKD	-	1626 (n) (67.67%): 15–29 ng/mL	-	-
	age 71.5 ± 11.7 yrs		777 (n) (32,33%): <15 ng/mL		
	33% men, 67% women				
Wahl [62] (2012)	1820 (n) DM with eGFR 40.7 ± 12.8 mL/min/1.73 m^2^	-	1820 (n)	-	-
	age 59.5 ± 9.8 years		23.9 ± 13.3 ng/mL		
Peng [63] (2015)	144 (n) T2DM and eGFR 45.2 (40.3-53.2) mL/min/1.73 m^2^	-	144 (n): 8.5 (6.8-11.3) ng/mL	-	-
	age 65 (IQR 52–75) years,				
	65.3% men, 34,7% women				
	median diabetes duration 14.5 (IQR 9.5–19.0) years				
Sipahi [64] (2016)	1463 (n) T2DM and CKD	-	6 (n) (0.8%) (stage 4) <20 ng/mL	239 (n) (31.9%): <20 ng/mL	446 (n) (59.5%): <20 ng/mL
	age 14–88 years		1 (n) (0.3%) (stage 4): ≥20 and <30 ng/mL		
	37% men 63% women		59 (n) (7.9%) (stage 3) <20 ng/mL	112 (n) (31.9%): ≥20 and <30 ng/mL	212 (n) (60.4%): ≥20 and <30 ng/mL
	serum level of Vitamin D <20 ng/mL reported in 770 (52.0%)		25 (n) (7.1%): ≥20 and <30 ng/mL (stage 3)		
	serum level ≥20 and <30 ng/mL in 357 (24.0%) patients.		23 (n) (7.1%) (stage 3): ≥ 30 ng/mL	106 (n) (32.9%): ≥30 ng/mL	193 (n) (59.9%): ≥ 30 ng/mL
Xiao [70] (2016)	240 (n) with T2DM, persistent microalbuminuria (AER 30–300 mg/24 h) or macroalbuminuria (AER >300 mg/24 h)	60 (n): 7.74 ± 2.90 ng/mL	60 (n) (stage 4): 8.44 ± 2.53 ng/mL	60 (n): 12.23 ± 4.07 ng/mL	-
			60 (n) (stage 3): 10.31 ± 3.36 ng/mL		
Ray [71] (2017)	72 (n) with “DM”	30 (n): 10.95 (IQR 9.3, 16.4) ng/mL	42 (n) (stage 4):19.15 (IQR 13.6, 23.4 ng/mL)	-	-
	age 54.2 ± 11.7 years				
	44 men, 28 women;				
	24.2% of CKD subjects were vitamin D deficient (<10 ng/mL)				
	41.4% having vitamin D insufficiency (10–20 ng/mL).				

(CI) Confidence interval, (CKD) chronic kidney disease, (eGFR) estimated glomerular filtration rate, (DN) diabetic nephropathy, (DM) diabetes mellitus, interquartile range (IQR), (n) number, (Yrs) years.

**Table 4:** Synthesized findings from recent trials on vitamin D dosing regimen.

	Population (n)	Intervention (6 Months)	Main Outcomes
De Zeeuw [98] (2010)	281 (n)	1 μg/d pct or 2 μg/d pct (oral)	UA reduction in 1 μg pct group from 613 to 554 mg/24 h [(95% CI −10% (−25 to 6); change −10% (−25 to 6)];
	age > 20 yrs		between-group difference –2% (95% CI −23 to 25; *p* = 0.86) with placebo group
	T2DM, albuminuria,		UA reduction in 2 μg pct group from 717 to 463 mg/24 h [(95% CI −34% (−45 to −21)];
	receiving RAAS inhibitors		between-group difference of –28% (95% CI –43 to –8; *p* = 0.009) with placebo group.
	eGFR: 15–90 mL/min/1.73 m^2^		Change in UACR –14% (from 63 to 54 mg/mmol; 95% CI−24 to −1) in 1 μg pct group; CI 95% −20% (from 61 to 49 mg/mmol; −30 to −8) in the 2 μg pct group; 16% (from 62 to 51 mg/mmol;95% CI −24 to −9) in the combined pct groups
Tiryaki et al. [99] (2016)	98 (n)	0.25 mg calcitriol/d (oral)	UACR from 186.58 ± 22.22 to 142.72 ± 12.38 mg/g (*p* = 0.014)
	age >18 yrs	
	T2DM, DN, albuminuria	
	receiving RAAS inhibitors		UAGT/UCre from 12.96 ± 2.76 to 8.64 ± 2.24 mg/g (*p* = 0.012)
	eGFR > 60 mL/min/1.73 m^2^		
Liyanage [100] (2018)	42 (n)	50,000 IU (0.25 mL)/month cholecalciferol (IM)	UA from 169.4 (35.8) to 117.6 (45.2) mg/g [95% CI; -51.8 (−66.1–−37.5)].
	age >18 yrs		UACR reduction of 51.8 mg/g (95% CI; −66.1–−37.5)
	albuminuria, DN		PR from 14.64 (5.62) to 8.83 (4.81) pg/mL, [95% CI; -5.7 (−6.7–−4.6)].
	eGFR > 30 mL/min/1.73 m^2^		

(ARB) Angiotensin receptor blockers, (CI) confidence interval, (CKD) chronic kidney disease, (DM 2) type 2 diabetes mellitus, (DN) diabetic nephropathy, (eGFR) estimated glomerular filtration rate, (PCT) paricalcitol, (PR) plasma renin, (IM) intramuscularly, (RAAS) renin-angiotensin-aldosteron system, (ref) reference, (UA) urine albumin, (UACR) urinary albumin-to-creatinine ratio, (UAGT/Ucre) urinary angiotensinogen/urinary creatinine, (Yrs) years.

## Abbreviations

15PGDH: 15-hydroxyprostaglandin dehydrogenase; 25(OH)D: 25-hydroxyvitamin D; ACEI: Angiotension converting enzyme inhibitors; ACR: Albumin-to-Creatinine Ratio; APC: Activated Protein C; Calcidiol: 25-hydroxyvitamin D; Calcitriol: 1,25-dihydroxy vitamin D; CI: Confidence Interval; CKD: Chronic Kidney Disease; COX: cyclooxygenase; CYP27B1: 1-alpha-hydroxylase; DBP: Vitamin D Binding Protein; DN: diabetic nephropathy; EPCs: Endothelial Progenitor Cells; ESKD: end-stage kidney disease; FGF23: fibroblast growth factor-23; FMD: Flow-Mediated Dilatation; GFR: Glomerular filtration rate; HOMA-IR: Homeostatic Model Assessment for Insulin Resistance; ICAM-1: Intercellular Adhesion Molecule-1; MCP: Monocyte Chemoattractant Protein; NF-κB: nuclear factor-κB; NR1I1: nuclear receptor subfamily 1, group I, member 1; PECAM-1: Platelet-Endothelial Cell Adhesion Molecule-1; PTH: parathyroid hormone; PTPN2: protein tyrosine phosphatase nonreceptor type 2; RAAS: renin–angiotensin–aldosterone system; RAGE: Receptor of Advanced Glycation End; RANTES: regulated on activation, normal T cell expressed and secreted; RCT: randomized controlled trial; STAT5: signal transducer and activator of transcription 5; sVCAM-1: Soluble Vascular Cell Adhesion Molecule-1; UA: urine albumin; uACR: Urinary albumin-to-creatinine ratio; UAGT/Ucr: urine angiotensinogen/urine creatinine ratio; VCAM-1: Vascular Cell Adhesion Molecule-1; VDR: nuclear vitamin D receptor; VEGF: Vascular Endothelial Growth Factor; WMD: Weighted Mean Different.
